# Viral entry pathways: the example of common cold viruses

**DOI:** 10.1007/s10354-016-0461-2

**Published:** 2016-05-12

**Authors:** Dieter Blaas

**Affiliations:** Max F. Perutz Laboratories, Department of Medical Biochemistry, Medical University of Vienna, Vienna Biocenter, Dr. Bohr Gasse 9/3, 1030 Vienna, Austria

**Keywords:** Endocytosis, Rhinovirus, Genome release, Lysosome, Uncoating, Endozytose, Rhinovirus, Genomfreisetzung, Lysosom, Kapsidöffnung

## Abstract

For infection, viruses deliver their genomes into the host cell. These nucleic acids are usually tightly packed within the viral capsid, which, in turn, is often further enveloped within a lipid membrane. Both protect them against the hostile environment. Proteins and/or lipids on the viral particle promote attachment to the cell surface and internalization. They are likewise often involved in release of the genome inside the cell for its use as a blueprint for production of new viruses. In the following, I shall cursorily discuss the early more general steps of viral infection that include receptor recognition, uptake into the cell, and uncoating of the viral genome. The later sections will concentrate on human rhinoviruses, the main cause of the common cold, with respect to the above processes. Much of what is known on the underlying mechanisms has been worked out by Renate Fuchs at the Medical University of Vienna.

## Zusammenfassung

Bei der Infizierung schleusen Viren ihr Genom in die Wirtszelle ein. Deren Nukleinsäuren befinden sich gewöhnlich gut verpackt innerhalb des Viruskapsids, welches wiederum häufig zusätzlich von einer Lipidmembran umhüllt ist. Beides schützt sie vor einer feindlichen Umgebung. Proteine und/oder Lipide auf dem Viruspartikel unterstützen die Anlagerung an die Zelloberfläche und die Internalisierung. Ebenso sind sie oftmals an der Freisetzung des Genoms in der Zelle beteiligt, welches als Vorlage für die Produktion neuer Viren dient. Im Folgenden werden die frühen, eher allgemeinen Schritte der Virusinfektion kursorisch dargestellt, dazu gehören Rezeptorerkennung, Aufnahme in die Zelle und Freisetzung des Virusgenoms. Im Hinblick auf die genannten Abläufe liegt der Schwerpunkt in den weiteren Abschnitten auf humanen Rhinoviren als Hauptursache für den Schnupfen. Viele der bisherigen Erkenntnisse in Bezug auf die zugrunde liegenden Mechanismen sind von Renate Fuchs, Medizinische Universität Wien, erarbeitet worden.

## Introduction

The first encounter between a virus and its host cell usually takes place via a protein, a proteoglycan, an oligosaccharide, or a glycolipid exposed on the cell surface that is recognized by cognate viral surface components. Only few viruses can subsequently penetrate directly from the plasma membrane into the cytosol of the host cell; the large majority rather exploits cellular entry pathways by travelling inside a membrane vesicle. Under physiologic conditions, these cell surface molecules serve as nutrient transporters or signal transduction receptors, or in cell–cell interactions and attachment, amongst other things. In the context of viral infection they have been termed “viral receptors” although the cell obviously does not make them for the purpose of becoming infected.

Viruses not only abuse membrane receptors but also soluble molecules, as a bridge to a plasma membrane protein or to bind to virus-specific antibodies that are present in the serum as a consequence of a previous infection. The latter, in turn, can then bind Fcγ-receptors present at the surface of some specialized cells and thus link the virus to them. For instance, virus-specific immunoglobulin G (IgG) can enhance infection by various flaviviruses, including, e.g., Dengue virus, rather than protecting the host against infection [1]. Other examples are the binding of apolipoprotein-E to both hepatitis C virus and heparan sulfate proteoglycans, which also leads to a connection between the virus and a cell surface component [2], and growth arrest-specific 6 (Gas6) bridging TAM receptors (various receptor tyrosine kinases) with phosphatidylserine that is present in the viral lipid membrane of Dengue and Vaccinia virus [3]. So, viruses have evolved to make use of whatever is available for attaching to and entering the host cell. Clearly, the presence or absence of a cognate viral receptor is a major but not the only factor in species and tissue tropism of a given virus [4]. It is of note that targeting viruses with oncolytic potential to tumors for their infection and destruction has been achieved by copying and exploiting this strategy [5].

Following the mutual recognition, the viral particle is taken up into the cell, either constitutively or triggered through its interaction with the receptor. The many identical subunits of a virion (i.e., a viral particle) render it multivalent; this can lead to clustering of, e. g., receptor tyrosine kinases or of integrins. For example, Vaccinia virus triggers activation of phosphatidylinositol 3‑kinase (PI3K)/Akt in an integrin β1-dependent manner, suggesting that this particular signaling pathway is essential for virus endocytosis [6]. Entry into the cell then engenders lipid vesicle trafficking processes. Reminiscent of the natural ligand, most intruding viruses become trapped where shallow membrane pits form; upon further membrane bending, a deep invagination is produced and finally severed from the plasma membrane in the form of a closed vesicle. The vesicle pinches off the inner side of the plasma membrane and carries its viral cargo, together with some extracellular fluid, through the crowded cytoplasm towards its intracellular destination. The transport vesicles undergo maturation by fusing with other vesicles, severing, and content sorting, all taking place during their voyage inside the cell. The vesicular membrane composition and intravesicular milieu continuously change along this route. Molecular machines, such as dyneins and kinesins, are involved in transporting virus-containing vesicles—or free virus particles—along actin fibers or microtubules [7–9].

Material inside the vesicle lumen is topologically extracellular and thus requires that either the entire virus or at least its genome penetrate the delimiting membrane to attain the cytosol. This occurs as soon as membrane composition, pH, ionic environment, etc. have become optimal for this process and a given virus; it can happen in early endosomes, late endosomes, recycling endosomes, macropinosomes, the endoplasmic reticulum, in various subcompartments of the Golgi, and even in lysosomes with their interior full of hostile hydrolytic enzymes.

Membrane penetration is usually preceded by or occurs in concert with conformational changes of viral surface proteins and/or the whole virion shell itself. In many cases, these structural changes require that an envelope protein, such as the hemagglutinin of influenza viruses, has been proteolytically cleaved during maturation [10]. A hydrophobic or amphiphilic fusion peptide then becomes exposed and inserts into the plasma membrane; in non-enveloped viruses, membrane destabilization or even disruption is brought about by the release of “membranolytic peptides”, small amphipathic viral proteins [11]. Another means of membrane destabilization was shown for canine parvovirus, which has a phospholipase A(2)-like domain in the N‑terminus of its capsid protein VP1, whose enzymatic activity makes the membrane permeable for dextran of 3 kD but not of 10 kD [12]. Recently, the perfidiousness of viral entry has been demonstrated for adenovirus; this virus induces small pores via its membrane lytic protein-VI, which triggers calcium-mediated lysosomal exocytosis repair pathways and lipid signaling that finally facilitates uptake of the pathogen [13]. In all these cases, once the nucleic acid(s) has arrived in the cytosol, depending on the type of virus and the nature of its genome, replication is either initiated right there, or after it has been shuttled to the nucleus.

The present article is aimed at providing a brief overview on these early events of viral infection. Its extent is far from covering all aspects of these complex and in part poorly understood processes. I apologize for citing only few, mostly recent and in part arbitrarily chosen publications referring to examples out of the about 17,500 papers found in a PubMed literature search for “virus AND entry” at the time of writing. For recent excellent and more in-depth reviews on viral entry and uncoating, see, for example, [14–23].

## Naked and enveloped viruses

**Fig. 1:**
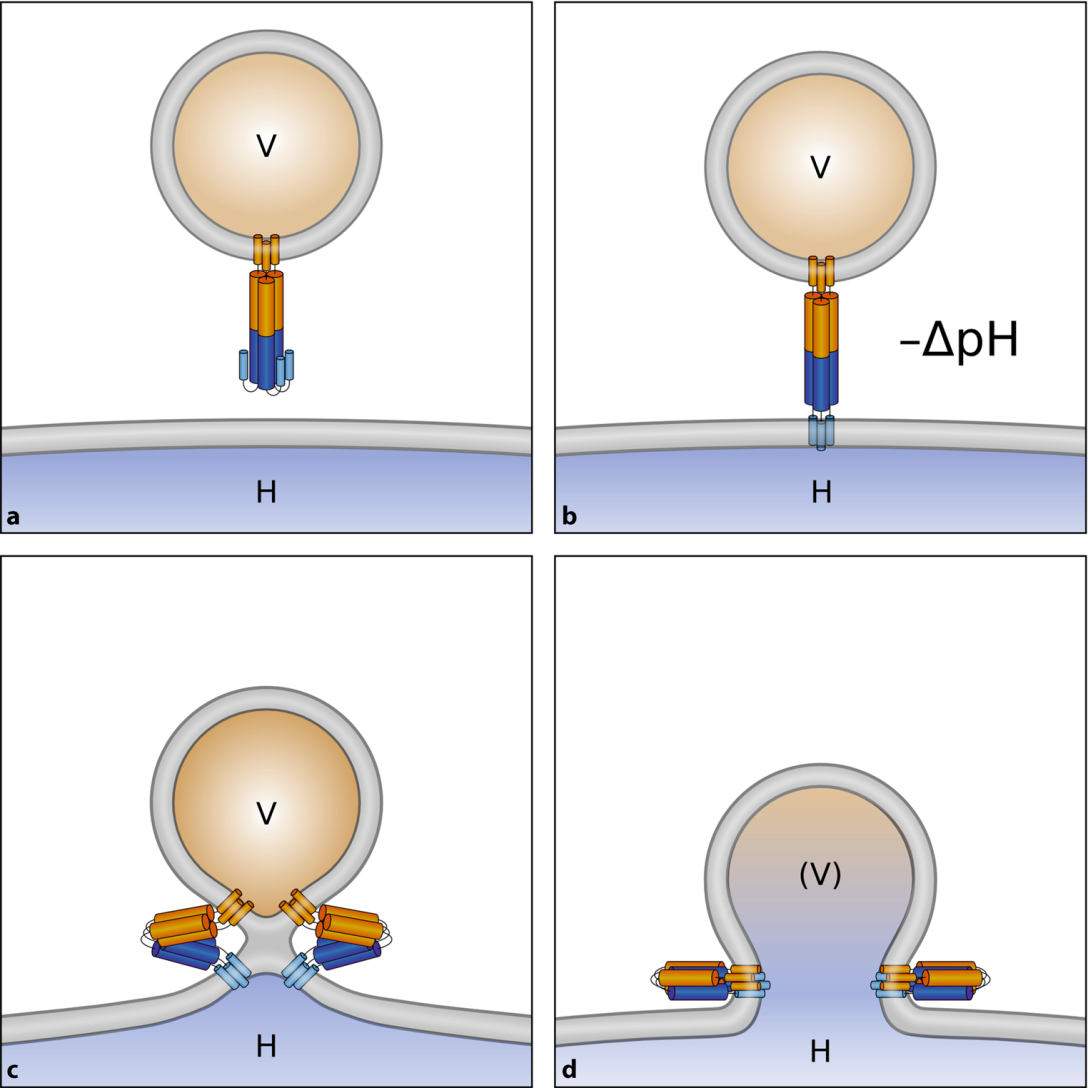
Fusion of enveloped viruses with a cellular membrane. **a** A viral envelope protein (often a trimer, as depicted) harbors a fusion peptide that is poorly solvent accessible. **b** On exposure to the acidic pH inside endosomes, structural changes occur that result in exposure of the fusion peptide and its insertion into the endosomal membrane. **c** Conformational rearrangements of multiple envelope proteins (just one trimer is shown) force the membranes into close apposition resulting in hemifusion without a fusion pore and only partial mixing of the lipids in one of the leaflets. **d** Complete fusion resulting in the nucleoprotein/nucleic acid (*brown*) accessing the cytosol. *V* virus, *H* host, *(V)* designates the residual viral membrane patch that remains after the contents have been transferred into the cytosol. Note that it finally becomes completely integrated into the host membrane with mixing of the lipids (not shown)

Viruses come essentially in two distinct flavors; either carrying a lipid membrane envelope decorated with viral proteins, or naked, i. e., without any lipid. The former have built-in machinery for penetration of their capsid into the cytosol via fusion of viral with cellular membranes. Most often, this process is triggered by the acid environment (pH between about 5 and 6) established inside the endocytic vesicles during their maturation into late endosomes [24–26]; there are only few viruses, including human immunodeficiency virus (HIV) and herpes simplex virus-1, which do not require an acidic environment and can thus even fuse with the plasma membrane at neutral pH [27, 28]. In any case, a structural change of a conformationally metastable viral surface protein needs to occur; this results in exposure of a previously hidden fusion peptide, usually a stretch of amphipathic and/or hydrophobic amino acid residues. The metastable state is often prepared during maturation via cleavage of the protein, setting the trigger for this sequence to insert into the cellular membrane upon arrival in a low-pH environment. The ensuing conformational change forces the viral membrane and the cellular membrane into close apposition. The result is hemifusion (i. e., an intermediate state without content mixing), and finally the complete melding of the two membranes, including lipid mixing and delivery of the nucleocapsid into the cytosol (Fig. 1).

Whereas membrane fusion is quite well understood, penetration of naked viruses still holds a number of open questions; in principle, access of the viral genome to the cytoplasm might either occur via disruption of the virus-containing vesicle—with the entire virion, together with other endosomal content, being released into the cytosol—or via pores of limited size in the endosomal membrane. Such pores are presumably lined by domains of viral proteins forming a channel contiguous with a pore in the viral shell. The holes in the virus capsid open upon a conformational switch, again most often triggered by the acidic pH and sometimes assisted by the viral receptor [29]. As described in more detail at the end of this article, by using common cold viruses as examples of cellular entry by non-enveloped viruses, Renate Fuchs has been working for many years on unravelling viral uptake and trafficking of viruses inside the cell, and the transfer of the viral RNA genome into the cytosol (see reviews [30, 31]). Because at least three different receptors are used by the three rhinovirus species, it is no wonder that they enter host cells via different endocytic pathways [32–34]. As a result, uncoating, i. e., release of their positive-sense single stranded RNA genome, might occur in different cellular compartments via different mechanisms, which is the subject of the last sections of this article.

## Viral attachment—role and function of the receptors

Parameters governing interactions between viruses and their cognate receptors have been investigated *in vitro* mostly by surface plasmon resonance methodology, for an example, see [35]. Virus is immobilized on a dextran-modified metal surface, a solution containing recombinant soluble receptor (i. e., lacking the membrane anchor) is constantly flown over the chip, and binding is monitored online as a mass-dependent change of the surface plasmon resonance angle. On washing with plain buffer, previously bound receptor dissociates, returning the signal to baseline. From these binding/unbinding profiles, the on and off rates and the affinity constants can be derived.

Capillary electrophoresis was demonstrated to yield information on the number of soluble receptors bound per virion by resolving virus with zero and up to 12 attached receptors. In addition, a rough estimate of the affinity constant could be derived [36]. Here, both components are free in solution and their concentrations and those of their complexes at different stoichiometry are measured.

Atomic force microscopy can deliver a detailed energy landscape of the unbinding forces when the virus is pulled away from the receptors and the (noncovalent) bonds between them are ruptured sequentially [37–39].

Finally, information on the spatial arrangement of the five ligand-binding modules of a soluble very-low density lipoprotein receptor (VLDLR) concatemer construct on the surface of a rhinovirus could be inferred from fluorescence resonance energy transfer between its N‑ and C‑termini. Quenching was recorded upon attachment. This demonstrated that the ends indeed come close to each other when the receptor molecules wrap around each of the vertices at the 5‑fold axes of icosahedral symmetry [40].

Although providing valuable information, the experimental conditions of at least the first two methods are not representative of the *in vivo* situation where the receptor is anchored in the cellular membrane*.* In this latter context, binding of a virus to its membrane receptor is not always as straightforward; it might be preceded by relatively unspecific electrostatic interactions with charged molecules at the cell surface, such as heparan sulfate or sialic acid, either tethering the virions to the plasma membrane and thus increasing their local concentration, or rather hampering their access to the receptor(s) and thus decreasing their local concentration [41]. Such effects are difficult to investigate; live-cell single-particle tracking methods [42] have suggested that some viruses remain on the cell surface for extended periods of time wandering. According to the “seek and stick” paradigm, a virus would thus diffuse in the plane of the membrane, bound weakly and rather unspecifically to main components of the glycocalyx, such as proteoglycans, glycolipids, etc., until it encounters its specific, higher-affinity receptor(s), usually present at lower density. Single receptor molecules might cluster during this diffusion in two dimensions, as a consequence of the multivalence of the virion. This can increase the avidity of the interaction, and, at the same time, slow down the speed of diffusion, i. e., the virus would collect several receptors on this excursion. In some specific cases, such avidity effects might turn on the endocytosis machinery at a site of multivalently attached virus by relaying signals into the cell [43]. There are also other ways one can envisage the sequence of events leading to formation of a virus–receptor complex ready to be engulfed; to give just one example, the high-affinity receptors might be confined to special microdomains (e. g., lipid rafts enriched in cholesterol), requiring the virus to first arrive in such microdomains by diffusion in order for tight binding to become possible [14]. High-speed particle tracking experiments have suggested that the plasma membrane is not a two-dimensional continuum fluid, but rather contains submicron compartments with different fluidity [44]. Reminiscent of signal transduction via bi- or oligomeric natural ligand molecules, the cytoskeleton might also be involved in limiting virus movements or in relaying viral entry via signals triggered through virus-induced clustering of the membrane receptors. It must be taken into account that the number of receptor molecules simultaneously attached to a single virion might impact on efficiency and speed of the uptake, as well as on uncoating later in the entry pathway, e. g., by exerting disruptive strain. For example, uncoating of HIV-1 was shown to depend on dynein and kinesin 1. It was suggested that these motors exert mechanical forces “tearing apart the virion” [45]. Another example is the pull of kinesin on adenovirus capsids when bound to the nuclear pore complex via Nup214 [46]. In addition, transport of receptor-bound virus to sites of active endocytosis (e. g., from filopodia to the cell body) has been observed.

## Uptake of the virus into the cell

Uptake of cargo, be it for nutrition, signaling, or for downregulating signals by ferrying receptor–ligand complexes to lysosomes for degradation, can occur mainly by i) clathrin-mediated endocytosis (CME), ii) caveolin-mediated uptake (CavME), iii) macropinocytosis, and iv), poorly characterized uptake mechanisms involving neither clathrin nor caveolin. These processes are usually summarized under the name of endocytosis. It is of note that often none of these pathways is exclusively exploited by a given virus; even more so when one entry route is blocked, e. g., by a specific inhibitor. In this situation, the cell might compensate by upregulating another pathway that will then also be used by the virus [47]. By the same token, a given virus might prefer different pathways in different cells [48].

Various components of the internalization machinery can be targeted by chemical inhibitors or ablated through genetic approaches such as RNA knockdown [49]. Recently, high-throughput screening of gene knockout libraries generated via CRISPR/Cas9 allowed identification of factors involved in uptake of bacterial toxins [50]; obviously, the same techniques can be used to find genes involved in viral endocytosis. Nevertheless, such results are sometimes ambiguous, either because of low specificity, redundancy of the targeted factors, or because the inhibited components take part in more than one pathway. Systematic and bioinformatics-guided RNAi screens have recently been adopted to identify proteins of the host cell involved in uptake of viruses [51–53]. This method involves cell transfection with short ‘interfering RNAs’ with complementarity to sequences of a target mRNA to be silenced. Their hybridization results in their degradation and consequently in downregulation of the encoded protein.

Finally, haploid mutant cell libraries—with each clone having a different gene inactivated—have been prepared. Screening such libraries for reduced viral replication has led to the identification of host components essential in virus uptake and to drugs specifically inhibiting the function of these factors [54, 55]. Such cellular components are clearly less prone to escape by viral mutation and are increasingly considered as possible targets for antiviral drugs.

## Clathrin-mediated endocytosis

**Fig. 2:**
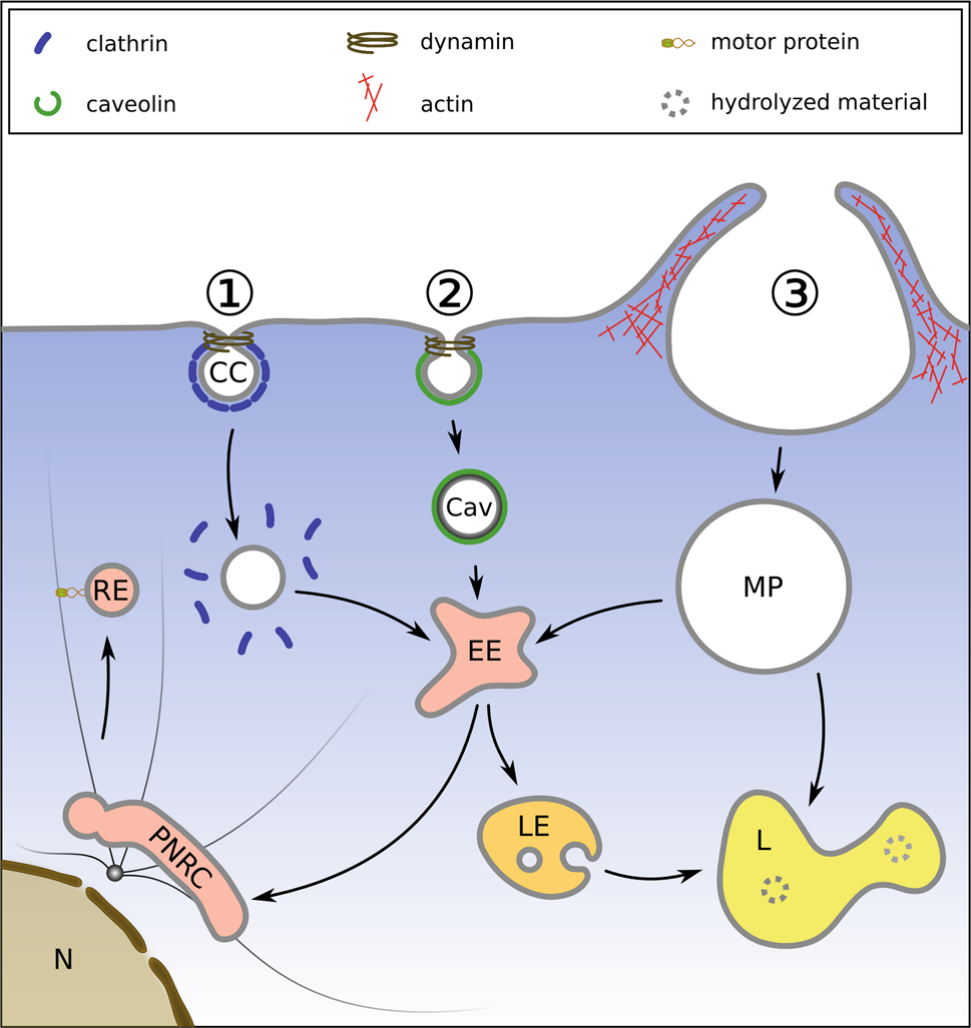
Simplified view of the major entry pathways. *1* Clathrin-dependent endocytosis. Clathrin-coated (*CC*) pits are formed at the plasma membrane and mature into clathrin-coated vesicles. These are severed from the plasma membrane by dynamin that forms rings around the necks. Once inside the cytosol, the coat is removed by uncoating ATPases, making the membrane accessible for fusion with other vesicles. Maturation and fusion results in the formation of early endosomes (*EE*); these mature further into late endosomes (*LE*) and/or fuse with LE. During the process, the pH continuously decreases from neutral to about 5.6, depending on the cell type. LE finally fuse with lysosomes (*L*), where the luminal content is degraded by hydrolases whose activity is maximal around pH 5. A side step from EE leads to the perinuclear recycling compartment (*PNRE*). Some ligands (e. g., transferrin) are returned to the plasma membrane via recycling endosomes (*RE*). *2* Caveolae (*Cav*) feature a particular lipid composition rich in cholesterol and glycosyl phosphoinositol-linked proteins, and a more translucent coat of cavin. They can be shuttled to endosomes but also to the Golgi (not shown here). *3* Macropinosomes (*MP*) form under the direction of actin fibers and transport extracellular liquid but also membrane-bound ligands. They travel to lysosomes for fusion and degradation of their content, but there is a connection to EE as well. Vesicles are often ferried along actin fibers and microtubules via motor proteins like kinesin and dynein (as indicated for RE)

CME (Fig. 2 pathway 1) is the by far best understood mechanism of cellular uptake (for an instructive movie see ref [56]). Many ligands (e. g., low-density lipoprotein, chylomicrons, Fe^3+^-saturated transferrin, growth factors, etc.) are channeled into this system via receptors whose cytoplasmic tails feature amino acid (AA) sequence motives like Asn-Pro-X-Tyr (X is any AA) or two consecutive leucines (the di-leucine motive) [57]. These signatures are recognized by the adapter complex AP-2 that tethers soluble clathrin monomers to the inner side of the plasma membrane for polymerization into a coat easily seen under the electron microscope. More than fifty other components are involved in CME; the membrane is curbed to build a shallow basket or pit that subsequently grows to form an invagination that becomes constricted and pinches off the plasma membrane. The main players in this process are the GTPase dynamin, the membrane-bending protein amphiphysin, and the AP-2 binding protein eps15 [58]. Finally, a clathrin-coated vesicle is formed [59]; once free in the cytosol, this coat is again removed under ATP hydrolysis, making the membrane accessible for fusion and fission with other vesicles [60]. During these maturation/fusion steps new proteins are acquired, amongst them the vesicular ATPase complex that acidifies the vesicular lumen by pumping H^+^ into the vesicle, creating an increasingly acidic intravesicular environment and a membrane potential [26]. The endocytic vesicles move on and finally fuse with lysosomes, the disassembly and degradation factories of the cell. Thus, viruses need mechanisms to release their precious genome undamaged into the cytosol to avoid destruction at the end of this itinerary by the aggressive lysosome.

CME can be blocked by various more or less specific chemical inhibitors, amongst them chlorpromazine and dynasore, by siRNAs (small interfering RNAs, see also RNAi above) knocking down the clathrin heavy chain and/or dynamin-2 (dyn2), and by expression of a dominant-negative form of this latter protein (dyn_K44A_) or of eps15 lacking the modules that interact with AP2 [61]. Chlorpromazine relocates clathrin and the adaptor complex AP-2 from coated pits to vesicles [62]; dynasore, the dyngos, dynols, and the iminodyns are all inhibitors of dynamin’s GTPase activity and, as such, target all processes involving dynamins [63, 64]. Pitstop inhibits interaction of amphiphysin with the amino terminal domain of clathrin and was thus expected to be specific for CME. However, it appears to also affect various forms of clathrin-independent endocytosis (CIE) [65]. Other measures, like potassium depletion or incubation of the cells with hypertonic sucrose, have been shown to dissociate polymerized clathrin and thereby inhibit entry of viruses by CME [66]. So, the best way to identify the main entry pathway exploited by a given virus is via employing several of the methods mentioned above in parallel.

## Caveolae-mediated endocytosis (CavME)

There are substantially fewer reports on viral entry involving caveolae than clathrin-coated pits, and only few viruses have been found to preferentially exploit this route. In addition, early reports on caveosomes, vesicles presumably derived from caveolae [67], in viral entry have been partially superseded by later results, e. g., see the case of echovirus 1 [68, 69]. Morphologically, caveolae are easily distinguishable from clathrin-coated pits; in the transmission electron microscope they show a typical elongated form reminiscent of round-bottom flasks extending into the cytosol, with a cavin coat that is less electron-dense than clathrin [70]. Their necks are open at the cell surface ([71]; Fig. 2 pathway 2). Apart from caveolin-1, a main component, caveolae possess a particular lipid composition with high cholesterol and glycosphingolipid content, and accumulate membrane proteins with glycosylphosphatidyl-anchors; as caveolae originate from cholesterol-rich lipid rafts, their lipid composition is similar. Viruses that might enter via this route include simian virus 40 (SV40), polyomavirus, echovirus 1 (but see above), certain coxsackieviruses, and some others [67]. Compared to CME, entry via caveolae is slow, i. e., the viruses might remain for hours unchanged in caveosomes, a distinct class of caveolin-1-containing endosomes identified in some cell lines. Similar to the reports on echovirus 1, SV40 entry in the absence of caveosome formation was also demonstrated. This questions the role of caveolin in the uptake of these particular viruses [72].

There are inhibitors that are, to some low extent only, specific for the caveolar pathway, such as the protein kinase inhibitor genistein. A combination of the cholesterol-sequestering drugs nystatin or filipin with the cholesterol-synthesis inhibitor progesterone blocks the formation of caveolae as summarized in [73]. Since severing of caveolae from the plasma membrane also involves dynamin, drugs blocking its function also block the caveolar pathway (see above).

## Macropinocytosis

During macropinocytosis cellular protrusions are actively formed and fold back onto the plasma membrane under the direction of actin assembly (Fig. 2 pathway 3; [74]); the process primarily takes up liquid but also material bound to the region where the vesicles form [75]. Few viruses have been shown to definitely enter via this pathway [76]; amongst them a murine amphotropic retrovirus [77], which this was inferred from its uptake into fibroblasts lacking caveolin or dynamin. Cytochalasin and latrunculin block both macropinocytosis and actin-dependent phagocytosis that is responsible for engulfment of larger particles, such as bacteria, and were shown to also reduce entry of this virus. Amiloride acts on Na^+^/H^+^ exchangers at the plasma membrane and has been used as a moderately specific inhibitor of macropinocytosis. At least in A431 cells it was shown to increase the submembranous pH that is normally lowered by metabolically generated acid; this, in turn, inhibits activation of GTPases involved in actin remodeling [78]. However, amiloride and it derivatives can also block later steps in viral synthesis, like replication of the nucleic acid; therefore, results with this drug need to be controlled with much care. The inhibitor profiles are often not exactly the same for the uptake of different viruses, making it difficult to term the process “typical” macropinocytosis. This might result from the virus exploiting other pathways in parallel to different degrees.

## “Virus-made invaginations”

The unique structure of the GM1 ganglioside, which can act as an SV40 receptor, together with the multitude of binding sites on the virion can promote membrane curvature and internalization by itself, in the absence of any invagination-promoting coats. Remarkably, even long tubules containing a multitude of viruses arranged as pearls on a string were observed [79]. Similarly, virus-like particles derived from a norovirus were shown to induce negative membrane curvature on binding to glycosphinoglipids present in giant unilamellar vesicles [80]. Although different from “pure” lipid-mediated endocytosis studied in liposomes [81], a similar effect might be responsible for deep invaginations seen around a rhinovirus (RV-A2) when bound to concatemers of five copies of repeat 3 of VLDLR, recombinant high-affinity protein receptors that were attached to liposomes via a his_6_-tag, which, in turn, was bound to Ni-nitrilotriacetate (Ni-NTA) lipids incorporated in the lipid bilayer [82]. Clearly, all cellular components involved in membrane-bending and fission were lacking in these latter highly artificial systems and no vesicles were observed inside the liposomes; formation of these pits and tubules might be strongly dependent on concentration, affinity, and size of the receptors. Similar tubules filled with intercellular adhesion molecule 1 (ICAM-1)-binding rhinoviruses had been observed in rhabdomyosarcoma cells overexpressing this receptor [33]. It is unknown whether a similar entry mechanism operates in vivo in cells expressing the respective receptors—protein but not lipid—at high concentration.

## Entry of common cold viruses—a paradigm for naked virus entry

Rhinoviruses (RVs) are the main cause of common colds. They are of enormous economic impact, estimated at about 25 billion USD per year in the USA due to absences from work, spending for medicines, and doctors’ visits [83]. Infections are recurrent and often involve different RV serotypes. Nevertheless, immunization with recombinant viral proteins seems to result in antibodies more broadly cross-reacting; however, again only with low cross-neutralization efficiency [84–87]. A major challenge for production of a vaccine is the large number of different viral serotypes, which would require the formulation to contain at least one representative of each cluster of the (weakly) cross-reacting antigens, which is difficult to realize.

## Rhinovirus receptors

RVs are of comparatively simple architecture; an icosahedral shell with T = 1, P = 3 symmetry is built from sixty copies of four different capsid proteins, VP1 through VP4, that enwrap a single-stranded positive-sense RNA genome of roughly 7100 bases in length. The particle is about 30 nm in diameter. Within the family picornaviridae, the *Enterovirus* genus includes more than 150 RVs divided into three species, RV-A, RV-B, and RV-C. It also includes the three poliovirus serotypes, the coxsackieviruses, and the well-known enterovirus EV71—causing epidemics of hand-foot-and-mouth disease with severe complications [88]—amongst many others. Despite high similarity of the nucleotide sequence of their RNA genomes [89] and three-dimensional structures, RVs recognize three different classes of receptors; twelve RV-A use members of the low-density lipoprotein receptor (LDLR) family for cell entry and constitute the minor receptor group [90–93]. Ninety species, comprising representatives of both species A and B, the major receptor group, bind ICAM-1 [94]. And finally, about fifty RV-C might bind the cadherin-related family member 3 (CDHR3), as so far explicitly demonstrated for three representatives [89, 95, 96].

The different receptors are unrelated and exhibit different AA sequences, 3D-structures, and functions in the context of cell metabolism. Whereas LDLRs are ubiquitous and highly conserved throughout animal species, ICAM-1 is not expressed in all cell types and is less conserved; major-group RVs only bind the human and primate version of ICAM-1. Systematic humanization of the mouse protein allowed pinpointing AA residues important for recognition [97]. The natural human ICAM-1 Kilifi variant substantially differs in affinity for RV14 and RV16 [98], allowing for estimation of the impact of a single AA mutation on virus recognition. Whereas numerous 3D-structures of virions belonging to the major and minor receptor groups in complex with their cognate receptors are available, most of what is known on CDHR3 interaction with HRV-Cs is derived from homology modelling [99] and no 3D structure is yet available. Probably, this is due to the low yield of virus in cells transfected to express the receptor; no established cell line that would become infected by RV-Cs has been found so far.

Members of the LDLR family possess various numbers of highly conserved ligand binding modules, seven in LDLR, eight in very-LDLR (VLDLR), and 31 in LDLR-related protein (LRP) [100]. Single modules exhibit very low binding affinity; however, simultaneous binding of several modules results in a considerable increase in avidity [101, 102]. Although not anticipated in initial cryo-EM work [103], a recombinant concatemer of five copies of module 3 of VLDLR was later demonstrated to arrange around a five-fold symmetry axis of the virus in a ring-like structure [40, 104, 105]. Interestingly, binding of a recombinant concatemer (i.e., five identical repeats fused head to tail) of repeat 3 of VLDL neutralized infectivity not only via competition with the natural receptor, but also via inhibiting the structural changes necessary for the release of the RNA [102, 106, 107]. Mutations of single VLDLR modules and their display on phage [108], as well as systematic exchange of human LDLR repeats for their mouse homologues [109] allowed the requirements of particular AA residues for viral recognition to be defined. The different modules in LDLR and LRP exhibit various degrees of affinity; therefore, they most likely contribute to the overall binding avidity to a different extent. The footprint of the receptor modules indicates that the interactions are mostly governed by charge and shape complementarity [90, 105, 110]. Since VLDLR is probably not expressed in the nasal mucosa [111, 112], presumably only LDLR and LRP are used by minor-group RVs for cell entry and infection in vivo [92]. In contrast to ICAM-1 (see below), LDLRs merely function as vehicles for virus entry. However, they might have a role in release of the virus from its receptors in the late endosomal compartment through the pH-dependence of the binding affinity [113].

LDLR molecules wrap around each of the 12 star-like mesas at the five-fold axes by engaging several, presumably up to five, ligand-binding repeats [104, 105]. In LRP, the 31 repeats are arranged in groups with spacers in between. Thus, it is possible that LRP can bind simultaneously to more than one pentamer because of its length. At maximal occupancy the stoichiometry (virus: soluble receptor) is 1:12 for recombinant VLDLR and, presumably, LDLR [113–116], but maybe less for LRP.

ICAM-1, the receptor of major-group RVs [117], belongs to the immunoglobulin (Ig) superfamily and has five Ig-domains [118], with only the first one (D1) being engaged in RV binding [119]; the second domain appears to be necessary for correct folding of D1. Cryo-EM reconstructions of complexes between representative RVs and soluble ICAM-1 show the latter sticking out from the canyon, upright with a slight outward inclination [120]. The theoretical stoichiometry at maximal occupation is 1:60 for ICAM-1 [121].

According to the canyon hypothesis, receptor-recognizing AA residues would be inaccessible for antibodies due to the narrowness of the canyon [122, 123]. Nevertheless, at least one monoclonal antibody (MAb) reaching far into this valley has been isolated. The paratopes of IgG molecules and of ICAM-1 are quite different and AA residues recognized by the MAb differ from those recognized by ICAM-1. This allows for escape from antibody neutralization via mutation without substantially changing receptor binding [124]. This also explains why ICAM-1 binds many different RVs, whereas MAbs targeting residues at the canyon floor and cross-reacting with several RV serotypes have not been described. On the other hand, for minor-group viruses, the receptor-binding site is entirely exposed and available for antibody recognition. Again, no cross-reacting antibodies targeting residues within the receptor footprint have been reported; in this case, this might be due to the necessity to bind more than one symmetrically-related site for appreciable avidity, which is impossible for IgGs because of their lower valence, geometry, and steric constraints. It has not been addressed whether IgMs, which would at least allow pentavalent attachment to such sites, might be better cross-reactors, assuming that the geometry and flexibility would allow such interactions.

A particular feature of ICAM-1 is its “catalytic” activity; in vitro, interaction with cognate RVs at a temperature above 30 °C leads in some, but not all, major-group RVs to uncoating at neutral pH, a process remotely similar to catalysis [121, 125–127]. Since initial low-affinity binding is followed by an increase in affinity it is likely that the receptor stabilizes an “open” conformation temporarily adopted by the virus via “breathing” [127]. This might allow ICAM-1 to penetrate more deeply into the canyon [128]. Breathing is also responsible for the transient exposure of capsid-internal protein sequences at the N‑terminus of VP1 and the myristoylated VP4 that are not accessible at lower temperature [129, 130]. Thus, ICAM-1 can be seen as a wedge driven into the canyon just when it is expanded. This would shift the equilibrium between the two conformations towards the more “open” one. The “priming” for uncoating strongly depends on the temperature and the concentration of soluble ICAM-1, which agrees with the dynamic structural rearrangements [131, 132]. However, receptor-driven uncoating is not very efficient at neutral pH but is aided by acidification [133]. RV-A89 adapted to grow in cells devoid of ICAM-1 was more readily neutralized by soluble ICAM-1. This might indicate that a decrease in stability is necessary for becoming less dependent, and finally independent—like minor-group RVs—from the destabilizing activity of ICAM-1 and allowing the use of a receptor that “just binds but does not uncoat” [134]. The concentration range of soluble ICAM-1 required for 50 % neutralization of different serotypes at physiologic pH extends over about 1.7 logs [135]; whether competition with the cellular receptor or ICAM-1-catalyzed uncoating is primarily responsible for neutralization cannot be inferred from these simple measurements. It is noteworthy that dimerization of the ICAM-1 molecule increases its neutralization potency [136], which might be a mechanical phenomenon resulting from generation of strain upon bivalent binding with suboptimal geometry. The “hit-and-run” uncoating of poliovirus [137] and EV71 by monoclonal antibodies [138] might be related phenomena.

The ratio between the number of physical rhinovirus particles and the number of infectious particles is in the range of 24–240 under mild purification conditions using metrizamide density gradients, and can be much higher when harsh conditions such as CsCl-gradient centrifugation are used [94]. It is not known whether this ratio is lower for unpurified virus. It is thus necessary to not only follow the physical particles on their way into the cell but also to demonstrate that a given pathway leads to initiation of infection. Therefore, it is essential to assess “productive” infection as well. Preferentially, such assays should target an early stage of the viral replication cycle to avoid secondary effects of inhibitors and/or particular experimental incubation conditions on later stages such as RNA replication, assembly, maturation, and release of progeny. Considering that i) uncoating results in release of the viral RNA into the cytosol, that ii) this RNA is translated, and that iii) the resulting polyprotein is cleaved to release the 2A protease, which, in turn, cleaves  the eukaryotic initiation factor 4G (eIF4G), the latter can be correlated with uncoating. The more RNA arrives in the cytosol the more eIF4G is cleaved. The tiny amounts of the viral proteinase P2A that becomes translated from the incoming viral RNA have thus been used to demonstrate viral uncoating without having to wait until replication has set in [33].

## Uptake and uncoating of the minor receptor-group prototype RV-A2

**Fig. 3:**
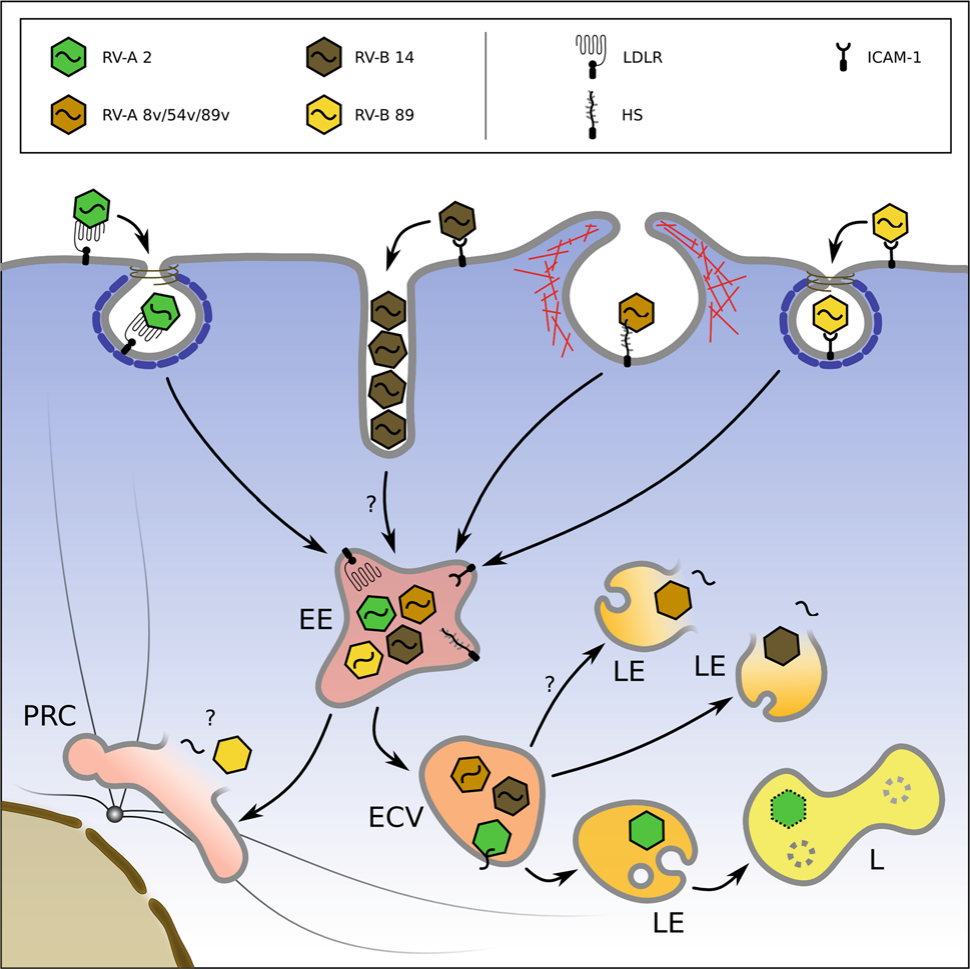
Simplified view of the entry of major receptor-group rhinoviruses binding ICAM-1 (RV-B14, RV-A89), the minor receptor-group rhinovirus binding LDLR (RV-A2), and the heparan sulfate-binding variant of the major-group virus RV-A8v. On attaching to their respective receptors, RV-A2 and RV-A89 are taken up into coated and RV-B14 and RV-A8v into non-coated vesicles and tubules. RV-A2 is shuttled to early endosomes where the pH is about 5.8 in HeLa cells. On further acidification to a pH below 5.7, RV-A2 releases the RNA through a pore and the remaining capsid proteins are transferred to lysosomes for degradation. RV-A8v enters by a pathway not involving clathrin in macropinocytic vesicles but also tubules (not shown); whether it releases its RNA through holes in the membrane or via lysis of the endosomes is not known. RV-B14 lyses the endosomal membrane and RNA, as well as viral protein, arrives in the cytosol. In this case, no lysosomal degradation is observed. RV-A89 travels to recycling endosomes for uncoating, whether it disrupts the endosomes is not known. *LDLR* low-density lipoprotein receptor, *ICAM-1* intercellular adhesion molecule 1, *EL* early endosome, *LL* late endosome, *PNRC* perinuclear recycling compartment

LDLR and LDLR-related protein 1 (LRP1) possess internalization sequence motives in their N‑terminal cytoplasmic tails that typically associate with the AP-2 complex, which, in turn, assembles the clathrin coat at the cytoplasmic side of the plasma membrane giving rise to clathrin-coated pits. Indeed, uptake of the minor receptor-group prototype RV-A2 into HeLa-H1 cells was demonstrated to be impeded by most inhibitors and interventions generally accepted to block CME, as summarized in Tab. 1 and Fig. 3. However, as mentioned above, endocytosis of RV-2A was also seen to be diverted to a non-clathrin-dependent route when the former pathway was curtailed by overexpression of the dominant negative dynamin 1 mutant K44A [139]. So, at least in HeLa-H1 (and in Rhabdomyosarcoma cells), RV-A2 primarily, but not exclusively, enters via clathrin-dependent endocytosis [33, 140].

**Tab. 1:** Compilation of data referenced in the text. Upper panel, impact of pharmacological inhibitors, overexpression of dominant negative mutant proteins, or other manipulations on viral entry either tested via immunofluorescence microscopy (IFM) or cleavage of eIF4G and/or replication by wildtype RVs and variants binding heparan sulfate, as exemplified by RV-A8v. Note that the inhibitors of proton carriers and weak bases increasing the pH in endosomes—such as niclosamide, monensin, methylamine, etc.—inhibit uncoating of minor-group RVs strongly and of some major-group RVs weakly. Lower panel, colocalization of viruses with various markers observed by fluorescence microscopy. For a list on the mode of action of these inhibitors see [49]

Serotype/receptor group	RV-A2/minor-group		RV-B14 (major-group), entering via ICAM-1		RV-A8v & other major-group entering via HS	
Process inhibited/tested via	Entry (obs. via IFM)	eIF4G clv./replication	Entry (obs. via IFM)	eIF4G clv./replication	Entry (obs. via IFM)	eIF4G clv./replication
*Inhibitor*						
Chlorpromazine	Not done	Strong	Not done	Marginal	Not done	No
Filipin or nystatin	Not done	No	Not done	No	Not done	No
Methyl-ß-cyclodextrin	Strong	Weak	Not done	Weak	Not done	Weak
Amiloride or EIPA	No	Strong	Not done	Strong	Not done	Strong
Dynasore	Strong	Strong	No	Weak	Not done	Strong
Cytochalasin D	No	Weak	Not done	Weak	Not done	Strong
Bafilomycin	No	Strong	Not done	Weak	Not done	Not done
Amphi-SH3	Strong	Not done	No	Not done	No	Not done
AP180-C	Strong	Not done	No	Not done	No	Not done
DynK44A	Strong	Not done	No	Not done	Strong	Not done
K-depletion	Strong	Not done	Not done	Not done	Not done	Not done
Rab5 S34N	Strong	Not done	Not done	Not done	Not done	Not done
*Colocalization with*						
Transferrin	Weak	Not done	No	Not done	No	Not done
Flotillin-1	Not done	Not done	No	Not done	No	Not done
CtxB	Not done	Not done	No	Not done	No	Not done
FITC-dextran	Not done	Not done	Strong	Not done	Strong	Not done
GFP-clathrin	Not done	Not done	Not done	Not done	No	Not done
Caveolin-1	No	Not done	Not done	Not done	No	Not done

List of abbreviations: *CtxB* choleratoxin B, *IFM* immunofluorescence microscopy, *Rab5 S34N* Rab5 with S34 mutated to N, *cvl* cleavage, *HS* heparan sulphate, *obs* observed

Once inside endosomal carrier vesicles (ECV) or late endosomes, RV-A2 is believed to dissociate from its receptor, a process partly promoted by the acid-dependent intramolecular competition of the beta-propeller domain of LDLR with the bound virus for the ligand binding domains [114, 115] and partly by the conversion of the native virus into the expanded A‑particle that lacks affinity for the receptor. Both effects occur at about pH 5.7, usually attained in ECVs and late endosomes, depending on the cell type [113, 141, 142], and are inhibited when the pH is increased by incubation with weak bases, proton carriers, and inhibitors of H^+^-ATPases [133, 143–145]. The affinity for the receptor is lost because of the altered conformation of the subviral particle within the receptor footprint [146, 147]. Presumably the particle is handed over to the membrane during its formation, a process assisted by the myristoylated VP4 and N‑terminal sequences of VP1 both exiting from the virion and inserting into the lipid bilayer creating pores lined by amphiphilic segments of these proteins [148, 149]. Flickering pores were detected by electrophysiological measurements in experiments with the related poliovirus [150–152] and with RV-A2 [30], and via the release of co-internalized FITC-dextran [153]. The amount of the dextran leaking from virus-containing endosomes into the cytosol was found to be inversely proportional to its size; whereas 10 kD dextran exited, 70 kD dextran did not. The membrane-disrupting adenovirus used as a control released both dextrans equally well. This led the authors to conclude that RV-A2 indeed formed pores in the endosomal membrane and that the RNA genome was most likely passed through such a pore into the cytosol. These results were supported by in vitro data demonstrating that the viral RNA could be transferred from membrane-bound virions into liposomes on acidification, and that the process was accompanied by membrane permeabilization but not disruption; the maximum RNA transfer occurred at a virus concentration that only marginally impacted on membrane integrity, again speaking for RNA transfer through pores of limited size [82]. Finally, *in vivo*, RV-A2 capsid proteins are readily degraded in lysosomes, indicating that the virus shell does not attain the cytosol. Proteolysis starts quite early; at about 30 min post infection, all of VP1 and a large proportion of VP2 had been cleaved [144]. Degradation of VP1 is inhibited by the microtubule-disrupting drug nocodazole, which blocks transport from ECV to lysosomes but leaves infection unaltered [143]. Nevertheless, it is possible that, similar to calicivirus infection [154], a marginal and thus difficult-to-detect cleavage of the subviral shell already occurs in (late) endosomes and facilitates RNA release in vivo.

Recently, it was demonstrated that RNA exit starts from the 3’-end on heating to 56 °C *in vitro* [155], as well as on physiologic endosomal acidification *in vivo* [156]. Interestingly, upon acidification *in vitro*, exit halted after about 700 bases had egressed. A similar phenomenon was seen *in vivo*; after conversion of the native virion into the subviral A‑particle, which was observed at about 5 min post infection at 34 °C, it took about 10 more minutes until the 700 3’-terminal bases became accessible to added nuclease; at lower temperature these times were prolonged. However, all the remaining genome sequences were lost from the viral shell at 34 °C within the next 2 min. It was also found that the partial egress *in vitro* quickly completed when a microsomal fraction prepared from HeLa cells was added. This suggests that cellular facilitators of the process might exist. Nevertheless, it remains enigmatic how an RNA molecule with a highly complex secondary structure with many stems, loops, and hairpins, should unfold to pass through a hole of about 0.1 × 0.2 nm in the absence of energy consumption in this short time.

## Uptake and uncoating of the major receptor-group prototype RV-B14

ICAM-1 lacks a clathrin localization signal but its cytoplasmic tail associates with alpha-actinin, thus connecting it to the cytoskeleton [157]. However, this association is not required for viral uptake since the tail and the transmembrane region can be replaced with a glycosylphosphatidyl anchor without impact on viral infection [158]. Therefore, it is not unexpected that chlorpromazine, which is moderately specific for the clathrin-dependent pathway, and some dominant negative mutant proteins involved in clathrin-dependent endocytosis failed to block RV-B14 uptake (Tab. 1 and [33]). A particularly striking observation was the accumulation of virions in long tubules connected to the cell surface in RD and BHK-cells expressing human ICAM-1 [33, 159]. Such aggregates had not been reported for viruses internalized by clathrin-dependent endocytosis, but were observed during entry of SV40 when bound to the lipid receptor GM1 ([79] and see above); nevertheless, in some instances, RV-B14 in vesicles with a coat looking very similar to a clathrin coat were seen in HeLa cells [159]. The significance of this observation for viral uptake is unclear. Entry was strongly reduced by the Na(+)/H(+) ion exchange inhibitor amiloride, and moderately reduced by the actin polymerization inhibitor cytochalasin. Thus, it is highly likely that RV-B14 enters by a non-clathrin- non-caveolin-dependent pathway resembling macropinocytosis. Entry is slower than that of RV-A2 and a large fraction of the virus appears to be retained at the plasma membrane despite of “normal” infection efficiency. As mentioned above, RV-B14 probably arrives in the cytoplasm as a whole (subviral) particle by disruption of the endosome [153, 160]; this contention was also supported by the finding that RV-B14 promotes endosomal release of transfection complexes [161]. In summary, RV-B14 enters via a pathway similar but not identical to typical macropinocytosis (Fig. 3).

## Uptake and uncoating of major receptor-group viruses adapted to heparan sulfate proteoglycan

Identical to RV-B14, the serotypes RV-A8, RV-A54, and RV-A89 all depend on ICAM-1 for entry and are thus major-group RV [32]. The latter three were adapted to use heparan sulfate (HS) as a receptor via blind passages alternating between HeLa and (ICAM-1-negative) Hep-2 cells. RV-A89 needed 34 such passages [134, 162], RV-A8 three [32], and RV-A54 was found to already be naturally adapted [34]. In all cases, binding to ICAM-1 was not lost but the viruses had become less resistant to moderately acidic pH. This suggests that in the absence of the “catalytic” activity of the receptor, the low pH alone must suffice for uncoating. HS-binding variants were shown to strictly depend on dynamin but not on clathrin, caveolin, and flotillin, as summarized in Tab. 1 for the HS-adapted variant RV-A8v, whose pathway was more extensively characterized; the same profile is probably seen with the other HS-adapted variants. The tabulated data show that entry via HS is similarly, but not identically sensitive to inhibitors and manipulations as entry via ICAM-1. Accumulation of the virions in tubules connected to the plasma membrane was also observed [32]. Recent work on uptake of wildtype RV-A89 (Conzemius et al. manuscript submitted) indicates that this major-group RV might involve clathrin for entry and uncoat in the endosomal recycling compartment (Fig. 3).

## Conclusion

Similar to the natural ligands of LDLR, minor-group RVs follow the clathrin-dependent pathway for entry. Once inside endosomal carrier vesicles or late endosomes, the low-pH environment triggers conversion of the native virion into the A‑particle, from which, in a poorly-understood process, the RNA is extruded through the endosomal membrane into the cytosol. Major-group ICAM-1 binding viruses appear to be taken up via a somewhat untypical form of macropinocytosis. Both pathways converge in early endosomes and the viruses are further shuttled to late endosomes. Aided by the destabilizing activity of ICAM-1 they are converted into A‑particles with concomitant disruption of the endosomal membrane, releasing the subviral particle or the empty particle together with already uncoated RNA into the cytosol. Finally, HS-adapted RVs follow a very similar pathway but behave similar to minor-group RVs in being stringently dependent on low pH for uncoating of the viral genome. Repeated attempts to adapt a major-group virus to use LDLR as a receptor failed. This indicates that recognition of LDLR is not limited to mere attraction of opposite charges. On the other hand, the ubiquitous HS can be exploited for attachment and entry but, at least *in vivo*, there must be other more stringent requirements for not using it in nature. The future might hold surprises regarding how RV-C viruses are taken up by CDHR3 and routed inside the host cell. Knowledge of how viruses enter the cell and points of possible intervention is of paramount importance for finding new ways of blocking infection with drugs. Cellular targets are increasingly considered for this purpose as this minimizes resistance via viral mutation.
